# Supplementary material for: Identification and functional analysis of non-coding regulatory small RNA FenSr3 in Bacillus amyloliquefaciens LPB-18
Source: PeerJ. 2023 May 15;11:e15236. doi: 10.7717/peerj.15236 (PMC10194069; doi:10.7717/peerj.15236)
Supplement: Supplemental Information 4 [file peerj-11-15236-s004.zip › KO/CK-vs-T1_map/map04911.html]

KEGG PATHWAY: Insulin secretion - Reference pathway


|  |  |
| --- | --- |
| **Insulin secretion - Reference pathway** |  |

[
Pathway menu
| Organism menu
| Pathway entry
| Show description
| User data mapping
]

Pancreatic beta cells are specialised endocrine cells that continuously sense the levels of blood sugar and other fuels and, in response, secrete insulin to maintain normal fuel homeostasis. Glucose-induced insulin secretion and its potentiation constitute the principal mechanism of insulin release. Glucose is transported by the glucose transporter (GLUT) into the pancreatic beta-cell. Metabolism of glucose generates ATP, which inhibits ATP-sensitive K+ channels and causes voltage-dependent Ca2+ influx. Elevation of [Ca2+]i triggers exocytotic release of insulin granules. Insulin secretion is further regulated by several hormones and neurotransmitters. Peptide hormones, such as glucagon-like peptide 1 (GLP-1), increase cAMP levels and thereby potentiate insulin secretion via the combined action of PKA and Epac2. Achetylcholine (ACh), a major parasympathetic neurotransmitter, binds to Gq-coupled receptors and activates phospholipase C- (PLC-), and the stimulatory effects involve activation of protein kinase C (PKC), which stimulates exocytosis. In addition, ACh mobilizes intracellular Ca2+ by activation of IP3 receptors.

|  |  |  |
| --- | --- | --- |
| Reference pathway | 184% 150% 122% 100% 82% 67% 55% | 图片下载 |
